# Opioids, sleep, analgesia and respiratory depression: Their convergence on Mu (μ)-opioid receptors in the parabrachial area

**DOI:** 10.3389/fnins.2023.1134842

**Published:** 2023-04-06

**Authors:** Nicole Lynch, Janayna D. Lima, Richard L. Spinieli, Satvinder Kaur

**Affiliations:** Department of Neurology, Division of Sleep Medicine, Beth Israel Deaconess Medical Center, Harvard Medical School, Boston, MA, United States

**Keywords:** analgesia, opioid induced respiratory depression, opioid use disorder, opioid tolerance, sleep-loss

## Abstract

Opioids provide analgesia, as well as modulate sleep and respiration, all by possibly acting on the μ-opioid receptors (MOR). MOR’s are ubiquitously present throughout the brain, posing a challenge for understanding the precise anatomical substrates that mediate opioid induced respiratory depression (OIRD) that ultimately kills most users. Sleep is a major modulator not only of pain perception, but also for changing the efficacy of opioids as analgesics. Therefore, sleep disturbances are major risk factors for developing opioid overuse, withdrawal, poor treatment response for pain, and addiction relapse. Despite challenges to resolve the neural substrates of respiratory malfunctions during opioid overdose, two main areas, the pre-Bötzinger complex (preBötC) in the medulla and the parabrachial (PB) complex have been implicated in regulating respiratory depression. More recent studies suggest that it is mediation by the PB that causes OIRD. The PB also act as a major node in the upper brain stem that not only receives input from the chemosensory areas in medulla, but also receives nociceptive information from spinal cord. We have previously shown that the PB neurons play an important role in mediating arousal from sleep in response to hypercapnia by its projections to the forebrain arousal centers, and it may also act as a major relay for the pain stimuli. However, due to heterogeneity of cells in the PB, their precise roles in regulating, sleep, analgesia, and respiratory depression, needs addressing. This review sheds light on interactions between sleep and pain, along with dissecting the elements that adversely affects respiration.

## Introduction

Physical pain is a guaranteed experience of just about any existence, yet opioids, one of the most effective treatments for pain, kills about 220 people per day in the United States (Centers for Disease Control Prevention [CDC], 2022). Initially declared an epidemic by the president in 2017, the opioid crisis has only gotten worse since then with opioid-related overdose deaths increasing by 44% in the following 3 years (National Institutes of Health [NIH], 2015). This is no surprise given that 1 in 5 people in the US, and globally, suffer from chronic pain (Goldberg and McGee, 2011; Yong et al., 2022). Used for thousands of years for pain and sedation, opioids first became commonly prescribed after new standards and regulations of pain management were established by the United States in 2000. Combined with incentives from the pharmaceutical manufacturing companies, and a lack of suitable alternatives, opioid prescribing quickly turned into over-prescribing (Jones et al., 2018). Significant research has since looked at opioids and the effect of prolonged use, finding that 1 in 4 patients receiving long term opioid treatment become addicted (Centers for Disease Control Prevention [CDC], 2017). Most commonly, long term users develop an opioid use disorder (OUD), which is a chronic relapsing disorder caused by intense cravings, increased opioid tolerance, and avoidance of withdrawal symptoms (Strang et al., 2020; Dydyk et al., 2022). The addictive quality of opioids have been attributed to the feeling of euphoria, in addition to analgesia, that the user experiences (White and Irvine, 1999). Over 16 million people worldwide, including 2.1 million in the US, suffer from an OUD of which 20% are estimated to eventually die from an overdose (Dydyk et al., 2022). Respiratory depression, specifically a decrease in respiratory rate and tidal volume, is ultimately what causes fatality from opioids (White and Irvine, 1999; Dahan et al., 2001; Pattinson, 2008). Overdose fatalities are common because, with repeated use, chronic opioid users gradually increase their dosage amounts (opioid tolerance) to achieve the same level of pain relief, slowly approaching amounts (White and Irvine, 1999) that depress all phases of respiratory activity (rate, minute volume, and tidal exchange) and produce irregular breathings (Pattinson, 2008; Dahan et al., 2010; Ramirez et al., 2021). Opioid analgesics including fentanyl depress respiration primarily by reducing the responsiveness of brain−stem respiratory centers to carbon dioxide (CO_2_) (Kirby and McQueen, 1986), therefore, opioids are also well-known to be associated with increased incidence of sleep-disordered breathing (SDB) pathology (Webster et al., 2008; Correa et al., 2015). Due to this, the infants less than 6 months old, opioid−naïve patients, the elderly, and those who have coexisting conditions such as chronic pulmonary disease and major organ failure, or are receiving other central nervous system (CNS) depressants, are some of the sub-populations that are at greater risk of opioid induced respiratory depression (OIRD).

In addition to respiratory depression, opioids also increase cardiovascular events, sleep disorders, clinical depression, hyperalgesia, risk of bone fractures, hormone dysregulation, immunosuppression, constipation, sedation, and dizziness among others (Baldini et al., 2012). In fact, sleep disruption (SD) occurs even in acute administration of opioids in healthy individuals, and contributes not only to the development of opioid dependence, but also relapse (Tripathi et al., 2020). A side effect with its own serious complications, sleep apnea is commonly experienced by opioid users and increases patients’ risk of coronary artery disease, heart attacks, heart failure, and strokes (Guilleminault et al., 2010; Schwarzer et al., 2015). On the contrary, treating OUD is in itself an effective treatment for sleep apnea, demonstrating the high interconnectivity of the opioid-induced analgesia and respiration pathways (Schwarzer et al., 2015). Further understanding of the bidirectional relationships shared between opioids, sleep, and respiration will help develop targeted therapies for pain management.

## Opioid receptors–their role in analgesia and respiration

Opioids act on opioid receptors, which are g-couple protein receptors (GPCRs) and are located throughout the body, both in the peripheral and central nervous system. There are four different opioid receptors that are structurally and functionally different. The delta (δ) opioid receptor, located mostly in the brain and mesenteric plexus, is responsible for spinal and supra-spinal analgesia, motor integration, thermoregulation (Mansour et al., 1988; Al-Hasani and Bruchas, 2011; Dietis et al., 2011). The kappa (κ) opioid receptor, in, spinal cord, and mesenteric plexus, produces analgesia, diuresis, food intake, neuroendocrine function and (Mansour et al., 1988; Al-Hasani and Bruchas, 2011; Dietis et al., 2011). The nociceptin opioid receptor (NOP), located in the spinal cord, depending on the concentration of opioids administered causes analgesia, hyperalgesia, and allodynia. Finally, the mu (μ) opioid receptor (MOR) derived from the Oprm1 gene, is located throughout the brain, spinal cord, mesenteric plexus, and submucosal plexus (Dahan et al., 2001; Dietis et al., 2011). The MOR is responsible for analgesia, sedation, respiratory depression, bradycardia, nausea, vomiting, and reduction in gastric motility. MORs alone are responsible for both pain and respiratory effects of opioids, which are the most adverse and clinically relevant effects of opioids for prescribing them as analgesics (Dahan et al., 2001). Other receptor subtypes show minimal effects on pain or respiration in the absence of MOR, therefore, this review will focus exclusively on the MOR (Meunier et al., 1995; Matthes et al., 1996).

## Opioid alternatives–blocking adverse effects of MOR agonists on respiration

Therapies that rescue respiratory depression following opioid administration are currently being investigated. Similar to Naloxone, the widely known non-specific opioid antagonist used to quickly reverse opioid overdose, non-opioid treatments like the potassium channel blocker (GAL021), ampakine (CX717), or 5-HT4(a) receptor antagonists have been shown to rescue opioid-induced respiratory depression, but not without affecting analgesia (Manzke et al., 2003; Oertel et al., 2010; Roozekrans et al., 2014). Though interventions are important to treat opioid overdose, alternative pain management therapies would be far more beneficial as they could replace opioids altogether. Novel GPCRs that primarily activate the G protein pathway with limited arrestin recruitment, as β-arrestin recruitment by the MOR appears to contribute to some of the unwanted effects of classical opioids (Mores et al., 2019), and therefore, these ligands are particularly interesting as potential targets for pain therapy. For instance, G-protein-biased μ-opioid receptor (MOR) activation using the ligands like TRV130, PZM21, and SR17018, may provide analgesia without the associated side effects of opioids including respiratory depression (Viscusi et al., 2016; Dahan et al., 2018; Elango et al., 2021). Additionally, use of poly-pharmacological ligands, which affect multiple receptor types that act on several different pain receptors or pathways, which could eventually summate to the same analgesic strength of opioids, without affecting respiration and sleep (Pasquinucci et al., 2021) is also promising. However, to accurately predict poly-pharmacology would also require high level of data curation, integration, and methodology development from various drug delivery disciplines. Peripherally restricted analgesics that target only peripheral opioid receptors, but not those in the central nervous system, could provide sufficient analgesia (Che and Roth, 2021) without the potential adverse effects. While these therapies seem promising, the lack of effective alternatives so far to opioids demonstrate the continued importance of further dissecting different elements of the pain and respiration pathways that are so much interwoven.

## Sleep loss/deprivation as a modulator of the opioid mediated analgesia

Numerous studies have investigated the impact of experimental sleep disturbances on pain perception (Moldofsky, 1995; Iaboni and Moldofsky, 2008; Finan et al., 2013; Schrimpf et al., 2015), and despite highly heterogeneous SD protocols, the overall results point to an increase in pain responses and behaviors when the sleep is disturbed in otherwise healthy humans, rats, or mice. Studies in rodents show that sleep loss either in the form of total sleep (Alexandre et al., 2020) or rapid eye movement (REM) sleep loss (Nascimento et al., 2007; Skinner et al., 2011) not only increases pain perception, but also decreases the analgesic efficacy of opioids. Furthermore, sleep apnea which is commonly experienced by opioid users, that also results in fragmented sleep, and may be an additional risk factor in developing opioid tolerance, OUD and susceptibility to relapse (Doufas et al., 2013; Jaoude et al., 2016; Ardon et al., 2020).

The loss of opioid efficacy in sleep-deprived individuals represents a potential major risk at the clinical level that could likely accelerate the development of tolerance, leading to dose escalation, and the risk of dependence or overdose. Interestingly, administration of the non-selective COX1/2 inhibitor ibuprofen failed to prevent both mechanical and heat hypersensitivity induced by 9 h of total sleep deprivation in mice and rats (Wodarski et al., 2015; Alexandre et al., 2020). This was reversed by caffeine and modafinil, two wake-promoting agents that have no analgesic activity in rested mice, but immediately normalize pain sensitivity in sleep-deprived animals, without affecting sleep debt (Alexandre et al., 2017). A similar study in rats also confirms an unexpected role for alertness in setting hyperalgesia (Hambrecht-Wiedbusch et al., 2017). Also studies in humans show that the increase in prostaglandin production, especially PGE2 is the potential mediator in sleep-loss induced changes in nociceptive processing (Haack et al., 2009; Simpson et al., 2020), and sleep loss produces an apparent loss of drug efficacy when compared to well-rested individuals. Therefore, there is an urgent need to identify the brain circuits that are primarily affected by sleep deprivation (e.g., over-activated wake circuits), which could be targeted to alter the efficacy of MORs in providing pain relief without producing opioid tolerance.

## Anatomical substrates for opioid induced respiratory depression (OIRD) and analgesia

To understand how pain and respiration pathways are interconnected, significant research has been done to determine which brain structures are important in facilitating respiration and, therefore, are most important in mediating OIRD. Both the analgesic and respiratory effects of opioid signaling are mediated by μ receptor 1 (Oprm1), which are inhibitory (Ramirez et al., 2021). The abundance of Oprm1 expression in the majority of the respiratory control areas of the brainstem has made it more challenging to resolve the neural substrates of respiratory malfunctions during overdose. Two main areas, the pre-Bötzinger complex (preBötC) in the ventral medulla and the PB complex in the upper brain stem area (Stucke et al., 2015; Miller et al., 2017; Varga et al., 2020) are implicated in regulating OIRD. The medullary ventral respiratory group (VRG) (Onimaru and Homma, 2006) that regulates inspiration and respiratory rhythmogenesis (preBötC) receives descending inputs from various areas including the PB and Kölliker Fuśe (KF) nuclei which exert significant additional regulatory control (Wang et al., 1993). Among the medullary VRG, only the preBötC is considered critical for depressive action of opioids (Mellen et al., 2003; Del Negro et al., 2018; Sun et al., 2019; Norris et al., 2021). In fact, additional research has shown that while the preBötC is responsible for respiratory rhythm generation, it is the PB, specifically, that has a modulatory effect, especially during OIRD (Eguchi et al., 1987; Bachmutsky et al., 2020). Further, MORs located in the PB, not the preBötC or KF, mediate the respiratory response to opioid administration as inhibition of the PB MORs mimic OIRD, while activation reverses it (Lalley et al., 2014; Liu et al., 2021). While the preBötC is important for generating respiration, it is the mediation by the PB that causes opioid-induced respiratory depression, making the PB, a structure of focus to dissect the elements that transmit pain, modulate respiration and sleep.

The PB, located in the rostral hindbrain at the midline of the pons and hindbrain, is split, medially and laterally, by a large fiber tract called the superior cerebellar peduncle (Palmiter, 2018). The PB has diverse neuronal populations that express MORs (PB^*Oprm*1^) and mediate normal breathing, affected by modified breathing during hypercapnia challenge (Kaur et al., 2017; Kaur and Saper, 2019, 2021) and by the OIRD (Hurlé et al., 1983). MOR-expressing neurons in the PB have been shown to be specifically important in cardiovascular, gustatory, and pain functions, which is understandable given their specific projection patterns to the amygdala, basal forebrain, amygdalo-piriform transition area, hindbrain reticular formation, and cranial motor nuclei (Chamberlin et al., 1999; Wilson et al., 2003; Huang et al., 2021a). Part of the initial difficulty in studying the role of PB MOR-expressing neurons (PB^*Oprm*1^) is their high levels of expression throughout the PB and that opiates can cause both sedation and wakefulness, depending on the site of action, receptor type, and dosage (De Andrés and Caballero, 1989).

Direct administration of opioid agonists locally into the PB results in suppression of respiratory rate, directly contrasting the results observed following local opioid administration within the VRG, particularly the preBötC (Krause et al., 2009; Mustapic et al., 2010; Stucke et al., 2015). In mice lacking the MOR, morphine-induced decrease in ventilation was abolished, suggesting that MOR is the site of action for respiratory effects of morphine (Dahan et al., 2001). In awake mice, removal of MORs from PB/KF neurons significantly rescues morphine-induced respiratory rate depression (Bachmutsky et al., 2020; Varga et al., 2020). This occurs at a therapeutically relevant analgesic dose and, importantly also at a very high dose that adversely affects respiration. By comparison, removal of MORs from preBötC neurons only rescues morphine-induced rate depression at lower doses, but at high doses further increases the occurrence of apneas (Varga et al., 2020). More evidence for the involvement of PB^*Oprm*1^ neurons in OIRD pathogenesis comes from a recent study, where it was shown that PB^*Oprm*1^ neuronal activity is tightly correlated with respiratory rate, and this correlation is abolished following morphine injection (Liu et al., 2021, 2022). Chemogenetic inhibition of PB^*Oprm*1^ neurons mimics OIRD in mice, whereas their activation following morphine injection rescues respiratory rhythms to baseline levels (Liu et al., 2021). This suggests that PB^*Oprm*1^ neurons may play a larger role in OIRD (Varga et al., 2020; Liu et al., 2021). However, their activation would inadvertently disrupt sleep and also increase pain sensitivity (Alexandre et al., 2017, 2020), as activation of most PB^*Oprm*1^ neurons promotes wakefulness (Kaur et al., 2013, 2017; Qiu et al., 2016; Kaur and Saper, 2019). Sleep disruption and increased pain sensitivity are major contributors to the development of increased opioid dependence, overuse and addiction (Hartwell et al., 2014; Koller et al., 2019; Huhn, 2021). Therefore, a better understanding of the PB neuronal subtypes is needed to prevent OIRD while preserving analgesia and sleep.

Opioids also reduce sensitivity to hypercapnic, hypoxic ventilatory responses, by acting on the MORs (Dahan et al., 2001; Pattinson, 2008; Lam et al., 2016) that possibly inhibit the PB^*Oprm*1^ neurons. Patients with opioid overdose lack hypercapnic arousal responses (Dahan et al., 2001; Pattinson, 2008; Lam et al., 2016). Under normal conditions, cessation of breathing during sleep (apneas) with obstructive sleep apnea (OSA) activates the respiratory chemosensory nuclei in the pons, namely, retrotrapezoid (RTN) and nucleus of solitary tract (NTS) that serve as relay centers for blood gas information that converges onto PB^*Oprm*1^ neurons, which promote arousal and also provide feed-back to respiratory control centers to increase respiratory drive. Distinct output pathways of the lateral PB neurons (dorsal vs. external lateral) may selectively affect the relay of the noxious input for mediating analgesic and sedative effects of opioids (Chiang et al., 2020). Calcitonin gene related peptide (CGRP) neurons in the external lateral part of PB (PB*^CGRP^*) are not only critical for relaying pain signals to the central nucleus of amygdala (CeA) (Neugebauer and Weidong, 2002; Han et al., 2015; Chen et al., 2017; Bowen et al., 2020) but this pathway may also transduce affective waking and cortical arousal in response to hypercapnia (Kaur et al., 2013, 2017; Qiu et al., 2016; Kaur and Saper, 2019).

## Heterogeneity in the PB

The neuronal subtypes in the PB are both diverse and intermixed (Geerling et al., 2011, 2016, 2017; Huang et al., 2021a; Yeghiazarians et al., 2021), a difficult combination when trying to determine the function of each in distinct sensory pathway. Prior research has relied on structural or location identifiers to distinguish different cell populations, which is now replaced by the use of the precise genetic markers that accurately identify the pivotal structure. Transcription factors like, Lmxb1 and Atoh1 mark two distinct developmental macro-populations (Karthik et al., 2022) in the PB, where the Lmxb1 population contains gene markers for FoxP2, Calca and Sat2b, while the Atoh1 contains pro-dynorphin (pdyn), g-protein coupled receptor (GPR) and FoxP2, and are located more ventrally (Karthik et al., 2022). Of those, Calca (encoding CGRP) has been linked to control over pain and hypercapnia induced arousal (PB*^CGRP^*). Fork head box protein transcription factor (FoxP2) expressing neurons (PB^*FoxP*2^) are located ventrally to that of the Atoh1 population, and are possibly associated with hypercapnia induced increase in respiration or with insufficient respiration as in OSA (Kaur and Saper, 2019, 2021; Karthik et al., 2022).

Advanced molecular tools provide more directed approach to dissect selective cell types. Use of targeted viral vectors (cre-dependent) and optogenetics, that specifically act on selective neuronal subtype and allow us to manipulate (activate or inhibit) them, while recording the animals for respiration, electroencephalogram/electromyography (EEG/EMG) signals, allows us to objectively investigate role of each cell types in pain, sleep and hypercapnia induced arousal (Kaur et al., 2013, 2017; Chiang et al., 2019, 2020).

**FIGURE 1:**
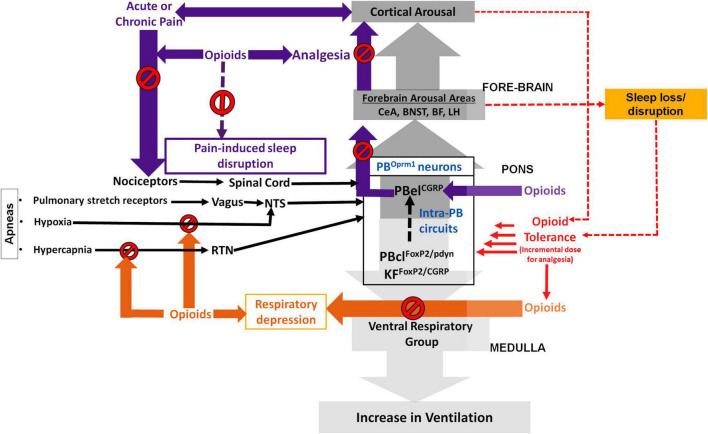
Schematic showing the possible role of the parabrachial μ-opioid receptor (MOR) expressing neurons (PB^Oprm1^) in regulating opioid induced respiratory depression (OIRD), analgesia, and sleep: The neurons in the centro lateral sub-nucleus of parabrachial area (PBcl) and Kölliker Fuśe (KF) that express FoxP2 (PBcl^FoxP2^) and those that express pro-dynorphin (PBcl^pdyn^) may critically modulate respiration through their descending projections to the medullary ventral respiratory group (VRG). Opioids at incrementally higher dose (due to developing opioid tolerance) may act by inhibiting the PBcl^FoxP2^/PBcl^pdyn^ neurons resulting in a continuous cycle of progressively depressed respiration that can prove to be fatal. Both hypercapnia and hypoxia stimuli (during apneic events) are conveyed to the PB *via* the retrotrapezoid (RTN) and nucleus of solitary tract (NTS), and these pathways are also inhibited by opioids use and tolerance. The interconnectivity of the PB^Oprm1^ neurons, specifically between the PBcl^FoxP2^/PBcl^pdyn^ and PBel^CGRP^ subpopulations may explain cortical arousal that results from respiratory stress during sleep apnea. The PB neurons that express CGRP (PBel^CGRP^) regulate waking up in response to pain, hypercapnia and aversive stimuli through their projections to the forebrain arousal areas such as, central nucleus of amygdala (CeA); the bed nucleus of stria terminalis (BNST); the basal forebrain (BF) and the lateral hypothalamus (LH). Opioids may provide analgesia and prevent sleep disruption (SD) by inhibiting the PBel^CGRP^ neurons that act as relay node for the pain stimulus which is transmitted to these neurons *via* the spinal cord. In contrast, inadequate sleep over-activate this cortical arousal circuit, inclusive of the PBel^CGRP^ neurons, which causes decreased sensitivity to inhibition from opioids, also known as opioid tolerance. Sleep disruption, in itself, also cause increased pain sensitivity, with decreasing levels of opioid induced analgesia. The resulting continuous cycle accelerates opioid tolerance while progressively reducing opioid analgesia.

The PB contains a population of glutamatergic neurons that are part of a chemosensory relay circuit projecting to forebrain arousal centers (Kaur et al., 2013; Yokota et al., 2015; Qiu et al., 2016; Saper and Kaur, 2018; Chiang et al., 2019). Calca-expressing neurons in the PBel (PB*^CGRP^*), which have the densest MORs expression in the PB (Wolinsky et al., 1996; Chamberlin et al., 1999; Miller et al., 2017; Huang et al., 2021b) regulate both pain-induced arousal (Kuner and Kuner, 2021) and respiration (Kaur et al., 2013, 2017; Qiu et al., 2016; Kaur and Saper, 2019). Thus opioid-induced inhibition of these neurons likely contributes to the sedative effects of opioids and can possibly explain their interconnected role in regulating pain, sleep and respiratory depression to opioids. Stimulation of PBel*^CGRP^* neurons affect cortical arousal, while their inhibition decreased hypercapnic arousal responses without affecting the ventilatory drive (Kaur et al., 2013, 2017, 2020; Kaur and Saper, 2019, 2021). In addition, the neurons located in the dorsal PB and caudal to the KF (that are FoxP2 or pdyn positive) also express Oprm1 (Chamberlin et al., 1999; Huang et al., 2021a). Our preliminary studies with manipulating and recording neuronal activity of the PB^*FoxP*2^ neurons shows that they are activated in response to hypercapnia, and further that their activity correlates with breathing and inhibiting these neurons reduces the minute ventilation in response to hypercapnia, suggests that PB^*FoxP*2^ neurons could likely mediate OIRD. Hypercapnia also activates a subset of FoxP2 neurons in the centro-lateral that express pro-dynorphin (PB*^pdyn^*). Thus, differential expression of Oprm1 on these three types of PB neurons (Huang et al., 2021a,b; Karthik et al., 2022) may contribute to the differential response to opioids, critical in defining respiratory depression and analgesic effects (Figure 1). Genetic silencing of PB^CGRP^ neurons also block pain responses and memory formation, whereas their optogenetic stimulation produces hyperalgesia aversive memory (Chen et al., 2017; Campos et al., 2018; Sun et al., 2020). PB^pdyn^ expressing neurons in the dorsal nucleus of the PB regulate body temperature (Norris et al., 2021) and also provide cellular substrate for transmission of nociceptive information to the PB^CGRP^ efferent (Chiang et al., 2019, 2020), which is important in pain processing.

Insomnia, pain induced sleep loss and apnea induced sleep fragmentation may cause over activation of the arousal circuits, that includes all the wake-active PB neurons (Figure 1) and their projection targets, which are usually inhibited by opioids to produce potent analgesia (Jasmin et al., 1994; Pieh et al., 2011; Hartwell et al., 2014; Jaoude et al., 2016; Bertz et al., 2019). In addition, opioids also enhance the inhibition of descending pain-modulating pathways contributing to anti-nociception (Millan, 2002; Lueptow et al., 2018). Sleep-loss alters these pathways as well altering the nociceptive processing, contributing to the development of opioid tolerance. Higher opioid doses further exacerbates sleep disturbances (O’Brien et al., 2021) increasing the risk for the associated respiratory depression.

With further increase in opioid-related deaths during the COVID-19 pandemic, it’s clear that the opioid crisis continues to be a significant health concern for unforeseeable future. Since sleep, respiration, and pain are so interconnected, pain-induced arousal and opioid-induced respiratory depression pathways possibly intersect at the PB, making this particularly vital to dissect its heterogeneous cell population and its role in regulating pain, respiration, sleep as well as their interaction with one another. This will also provide insight into the additive effects of alcohol use disorder, which may employ the same neuronal pathway, as is evidenced by growing research on the use of MOR antagonists to treat alcoholism (Drobes et al., 2003; Ripley et al., 2015; Ben Hamida et al., 2019). As opioids and alcohol both cause systemic depression, largely acting on MOR and GABA, respectively, to mediate inhibition (Chamberlin et al., 1999; Morrow et al., 2001; Ben Hamida et al., 2019). Frequent users of both alcohol and opioids also experience chronic pain and hyperalgesia which is further exacerbated by use of these substances together (Witkiewitz and Vowles, 2018).

More studies are required targeted at characterizing the role of different PB neuronal subsets in mediating the OIRD, which will help design better therapeutics that will prevent not only respiratory depression, but will also help spare opioid induced analgesia and prevent pain induced SD and fragmentation. Alternatively, given the bidirectional relationship between pain and sleep, treating the disturbed sleep may also be the key to preventing opioid overuse, withdrawal, poor treatment response for pain and addiction relapse.

## Author contributions

NL: conceptualization, research, first draft, and editing. JL and RS: research support and editing. SK: conceptualization, research, supervision, and editing. All authors contributed to the article and approved the submitted version.
